# Supplementary material for: Response of Guiana dolphins to the construction of a bridge in Ilhéus, Northeastern Brazil
Source: PLoS One. 2024 Nov 15;19(11):e0312476. doi: 10.1371/journal.pone.0312476 (PMC11567520; doi:10.1371/journal.pone.0312476)
Supplement: Attachment — Submitted filename: Comments in Manuscript.docx [file pone.0312476.s001.docx]

This manuscript by Pendu et al addresses an important aspect of the impact of structural interventions on riverine species. Overall, the manuscript read smooth, figures ad tables are well organized. I recommend this manuscript may be accepted for publication. Authors may consider to incorporate the following suggestions; I hope these may help to improve the quality of the manuscript.

In introduction, please review the similar studies conducted globally. For example, author can find a similar studies on the Indus and the ganga river river dolphins. You may find Sonkar et al 2020, where they have studied the impact of large barrages on the ganga river dolphins, a similar study has been conducted by Sinha et al as well.

Methods:

Please don’t keep study area and subjects under the methodology section. You can create a separate section for study area. If possible, use some satellite imager to show the condition of river before and after the construction of bridge. It will help readers to understand the change in the river patter or morphology due to the construction activities.

In method section, if possible, include a flow chart to illustrate the overall procedure followed in this study. I believe this will help reader to quickly understand the setps followed.

Discussion:

Here you discuss your findings in chronological order and try to explain the result with finer details.

Conclusion:

Please add a separate section for conclusion. And highlight the major findings in bullet.

I congratulate authors for this work. Hope to see this work published.
