# Supplementary material for: Response of Guiana dolphins to the construction of a bridge in Ilhéus, Northeastern Brazil
Source: PLoS One. 2024 Nov 15;19(11):e0312476. doi: 10.1371/journal.pone.0312476 (PMC11567520; doi:10.1371/journal.pone.0312476)
Supplement: Attachment — Submitted filename: Bridge construction affect dolphin.docx [file pone.0312476.s002.docx]

Manuscript entitled “**Do bridge constructions affect dolphin use of space? Responses of Guiana dolphins from Ilhéus, Northeastern Brazil**”. I have gone through the entire manuscript thoroughly.

This manuscript investigates the impact of the construction of the Jorge Amado Bridge on the space use and movement patterns of Guiana dolphins (*Sotalia guianensis*) in the Cachoeira River estuary (Pontal Bay) in Ilhéus, Brazil. The authors used theodolite and total station observations to track the dolphins' trajectories and estimate their utilization distribution, area of use, and core areas before and during the bridge construction.

**The key findings of the manuscript are:**

1. Contrary to the authors' expectations, the dolphins increased their use of the areas close to the bridge during its construction. This was likely due to the formation of a sandbank near the bridge, which may have provided new foraging opportunities for the dolphins.

2. The dolphins consistently avoided areas close to the river banks, both before and during the bridge construction, possibly due to the presence of debris and stranded boats.

3. The authors suggest that changes in the local bathymetry, caused by the bridge construction and the resulting sandbank formation, were the main drivers of the observed changes in the dolphins' space use.

**Scientific Observations:**

1. The introduction provides a comprehensive background on the potential impacts of coastal constructions on cetaceans, particularly Guiana dolphins, which are the focus of this study. The introduction is well-structured and effectively sets up the rationale for the study.

2. The methods section is detailed and the data analysis approaches are appropriate for the research questions. The use of theodolite and total station observations to track the dolphins' movements and estimate their utilization distributions is a robust approach.

3. The results are clearly presented, and the authors make good use of figures to illustrate the changes in the dolphins' space use before and during the bridge construction.

4. The discussion section provides a thoughtful interpretation of the results, considering both the direct and indirect impacts of the bridge construction on the dolphins' behavior and habitat use. The authors' hypothesis about the role of bathymetric changes in driving the observed patterns is well-supported by the data.

**A few of my specific comments and suggestions are as follows:**

**Comment. 1**

"What specific environmental changes associated with the construction of the Jorge Amado Bridge may have facilitated new foraging opportunities for Guiana dolphins, and how might these changes influence their long-term habitat selection and behavior in Pontal Bay?

Additionally, what other factors—such as prey availability, habitat complexity, and social dynamics—might influence dolphin behavior and habitat selection in response to anthropogenic disturbances?

**Comment. 2**

Line 42: Describe more about Guiana dolphins (*Sotalia guianensis*) like what functions paly in that ecosystem, How is it different from other dolphin species?, its ecological roles/ importance in Cachoeira River estuary.

**Comment. 3**

Line 84: Few description of under Construction Bridge should be included like, how much area was covered by the pillars of the bridge, what was the distance between the two pillars during construction? etc.

**Comment. 4**

Line 85: During the construction of the bridge, the width of the estuary mouth was reduced, and the sandbank expanded. This study was conducted in the estuary; have any tidal affect observed in the behaviors of dolphin population during the study period? Or what were the visiting / sighting frequencies during the variation in the depth of the estuary.

**Comment. 5**

Mention the table number in the entire manuscript

**Comment. 6**

Justify lines and correct some minor grammatical mistakes in the manuscript

Overall, this is a well-designed and executed study that provides valuable insights into the behavioral responses of Guiana dolphins to the construction of a major coastal infrastructure project. The authors' findings contribute to the limited knowledge on the impacts of anthropogenic activities on this vulnerable species and can inform conservation and management efforts.
