# Supplementary material for: Response of Guiana dolphins to the construction of a bridge in Ilhéus, Northeastern Brazil
Source: PLoS One. 2024 Nov 15;19(11):e0312476. doi: 10.1371/journal.pone.0312476 (PMC11567520; doi:10.1371/journal.pone.0312476)
Supplement: Attachment — Submitted filename: Review-Do bridge constructions affect dolphin use of space.docx [file pone.0312476.s003.docx]

I have gone through the manuscript- Do bridge constructions affect dolphin use of space? Responses of Guiana dolphins from Ilhéus, Northeastern Brazil, which looks at the impact of the construction of the Jorge Amado Bridge on the movement of Guiana dolphins. Changes in the site bathymetry due to bridge construction and the resulted sandbank formation, are the major reasons for the dolphin space use pattern.

Few comments:

It appears that Guiana dolphins forage in shallow water and estuaries and therefore, sand banks created due to bridge construction and other barriers facilitated herding of fishes by dolphin towards the barrier. It is an opportunistic foraging behavoiur. However, it imperative to look at the long-term impact of such barriers and adopted behaviour on the dolphin.

Authors would like to add such prospectives in their introduction as well as discussion. This will help in formulating long term planning for systematic monitoring of such projects which affects the critical aquatic lives.
