# Supplementary material for: Response of Guiana dolphins to the construction of a bridge in Ilhéus, Northeastern Brazil
Source: PLoS One. 2024 Nov 15;19(11):e0312476. doi: 10.1371/journal.pone.0312476 (PMC11567520; doi:10.1371/journal.pone.0312476)
Supplement: Attachment — Submitted filename: Response to Reviewers1.docx [file pone.0312476.s004.docx]

Response to reviewers

# PONE-D-24-14912

# Do bridge constructions affect dolphin use of space? Responses of Guiana dolphins from Ilhéus, Northeastern Brazil

# PLOS ONE

Dear Academic editor:

First of all, we are grateful for the interest of the journal PLOS ONE' in publishing our manuscript. This revision note is a detailed response to your helpful comments and those of the two independent reviewers and complements the revised version of the manuscript, for your consideration. We have taken all comments into account, and responses to each comment are organized by reviewer.

# Journal requirements:

When submitting your revision, we need you to address these additional requirements.

1. Please ensure that your manuscript meets PLOS ONE's style requirements, including those for file naming.

**Done**

2. In your Methods section, please provide additional information regarding the permits you obtained for the work. Please ensure you have included the full name of the authority that approved the field site access and, if no permits were required, a brief statement explaining why.

**We added the following sentence in the Data collection section: “No permits were required for this study, which was carried out in an unprotected area and involved the remote observation of animals in the wild.”**

3. We note that your Data Availability Statement is currently as follows: [All relevant data are within the manuscript and its Supporting Information files. Please confirm at this time whether or not your submission contains all raw data required to replicate the results of your study. Authors must share the “minimal data set” for their submission (…).

**We uploaded in Biostudies repository (Licence CCO, DOI: 10.6019/S-BSST1636) the following set of 4 excel tables corresponding to the data analyzed in the article to be published in PLOS ONE:**

**1.visitation rate: Visitation rate of Guiana dolphins in Pontal Bay.**

**2.bimonthly_overlap: Raw data used to analyze the degree of bimonthly overlap between areas of use and between core areas (HR95, HR50, UDOI95, UDOI50).**

**3.RSF_and_UD_diff_analysis: Resource Selection Functions and Utilization Distribution differences analysis.**

**4.velocity_and_linearity_analysis. Raw data used to analyze the impact of bridge construction on dolphin movement (speed and linearity)**

4. We note that Figures 1 and 3 in your submission contain [map/satellite] images which may be copyrighted. (…).

**We modified Fig 1: the map does not contain anymore original satellite image, only illustrative maps created by the authors. Fig 4 (formerly Fig 3) contains maps created by the authors, and we have mentioned the name of the author of the bridge photograph (Fig 4C) in the figure legend (Photo: Y. Le Pendu).**

5. Please review your reference list to ensure that it is complete and correct. (…).

**Done.**

**Response to academic editor comments:**

*Changes in the site bathymetry due to bridge construction and the resulted sandbank formation, are the major reasons for the dolphin space use pattern. Can the Ms be correlated with habitat chief of dolphins during Covid-19 lock down i.e. in contrary to bridge construction work (disturbance) undisturbed regime also affect home range of these river cetaceans. Some such explanation about bankcard shifting of Gangetic dolphins have been provided. in following paper: COVID-19 lockdown affects zooplankton community structure in dolphin appearing site of the River Ganga at Patna_26_1*

*August 2023Aquatic Ecosystem Health and Management 26(1):20-31*

**R: Although analyzing the influence of COVID-19 is certainly a promising direction for future research, our data collection was completed in February 2020, prior to the onset of the COVID-19 lockdown in our study area. The first confirmed case of the virus in Brazil was on February 25, 2020, and protective measures in Ilhéus began on March 6, which halted our sampling. Consequently, our results are not influenced by the COVID-19 lockdown, and addressing this is beyond the scope of our study. To clarify this, we have added the sentence, 'The sampling was completed prior to the COVID-19 lockdown in Ilhéus', to the Data Collection section, ensuring that readers do not misinterpret our findings.**

**Response to reviewer #1 comments:**

*This manuscript by Pendu et al addresses an important aspect of the impact of structural interventions on riverine species. Overall, the manuscript read smooth, figures ad tables are well organized. I recommend this manuscript may be accepted for publication. Authors may consider to incorporate the following suggestions; I hope these may help to improve the quality of the manuscript.*

*In introduction, please review the similar studies conducted globally. For example, author can find a similar studies on the Indus and the ganga river river dolphins. You may find Sonkar et al 2020, where they have studied the impact of large barrages on the ganga river dolphins, a similar study has been conducted by Sinha et al as well.*

**R: We added a sentence citing the review by Campbell et al. (2022) and the studies by Pavanato et al. (2016), Aggarwal et al. (2020): “Some studies indicate that dam construction can have serious consequences for river dolphins such as the boto (*Inia geoffrensis*)[16] and Ganges river dolphins (*Platanista gangetica gangetica*)[15,17], potentially leading to unsustainable population fragmentation and even local extinction [15]”. We also cite the study of Sonkar et al. (2020) in the sentence: “Similarly, Ganga River dolphins (*Platanista gangetica*) altered their preferred areas, increasing their occurrence in certain portions of the river after the construction of a barrage [18].”.**

*Methods:*

*Please don’t keep study area and subjects under the methodology section. You can create a separate section for study area.*

**R: We have renamed the "Study area and subjects" section to "Study area”. We moved the two sentences referring to dolphins [Groups of *S. guianensis* (…) consist of 2 to 5 individuals] to the beginning of the "Data collection" section. We hope to have contemplated your request.**

*If possible, use some satellite imager to show the condition of river before and after the construction of bridge. It will help readers to understand the change in the river patter or morphology due to the construction activities.*

**R: Figure 1 shows two illustrative maps based on satellite images depicting Pontal Bay before (A) and after (B) the completion of the Jorge Amado Bridge, in particular the evolution of the sandbank.**

*In method section, if possible, include a flow chart to illustrate the overall procedure followed in this study. I believe this will help reader to quickly understand the setps followed.*

**R: We added a new figure 2, a flowchart summarizing the stages of data collection, selection, organization and analysis carried out.**

*Discussion: Here you discuss your findings in chronological order and try to explain the result with finer details.*

**Correct**

*Conclusion: Please add a separate section for conclusion. And highlight the major findings in bullet.*

**R: A separate section for conclusion was added, with the major findings highlighted in bullets, although such conclusion in bullets does not seem to be a common practice in articles published in PLOS ONE. According to guidelines, “Results, Discussion and Conclusions may all be separate, or may be combined to create a mixed Results/Discussion section (commonly labeled “Results and Discussion”) or a mixed Discussion/Conclusions section (commonly labeled “Discussion”)”.**

**Response to reviewer #2 comments:**

*Comment. 1. "What specific environmental changes associated with the construction of the Jorge Amado Bridge may have facilitated new foraging opportunities for Guiana dolphins, and how might these changes influence their long-term habitat selection and behavior in Pontal Bay?*

**R: Thank you for this suggestion. We pointed out in the third paragraph of the discussion [The reduction in river flow...] that the formation of a sandbank near the bridge was the environmental change that facilitated new foraging opportunities, as it probably functions as a barrier that allows fish to be trapped. At the end of the third paragraph of the discussion, we have added the following sentence to address the long-term impact of environmental changes: “As the geomorphological and sedimentological dynamics of Pontal Bay are unpredictable, only monitoring the growth of the sandbank and the presence of dolphins will reveal whether the new opportunistic feeding area identified in this study will continue to attract dolphins or whether they will have to forage in other areas.”. Potential long-term effects are outlined at the end of the discussion: “We highlight that Pontal Bay has suffered continuous silting due to the deposition of continental and oceanic sediments.” (…) “****The Jorge Amado Bridge poses yet another threat that could reduce the depth and width of Pontal Bay over time, two environmental characteristics that could decrease habitat suitability for dolphins. A continuous monitoring of Guiana dolphins in this area is highly recommended to understand the long-term impacts and to proactively conserve this population.”**

*Additionally, what other factors—such as prey availability, habitat complexity, and social dynamics—might influence dolphin behavior and habitat selection in response to anthropogenic disturbances?*

**R: The multiplication of anthropogenic disturbances along the coastal zone can have an impact on dolphin behavior. However, the manuscript focuses on the impacts of bridge construction on the space use of dolphins in Pontal Bay, which is only part of the home range of these dolphins, that also feeds and socializes in the coastal waters of the ocean.**

*Comment. 2. Line 42: Describe more about Guiana dolphins (Sotalia guianensis) like what functions paly in that ecosystem, How is it different from other dolphin species?, its ecological roles/ importance in Cachoeira River estuary.*

**R: We added the following information to the text: (…) Guiana dolphins regularly visits (… Pontal Bay), mainly for foraging purposes [31]. It is the only dolphin species that frequents the estuary. As a top predator restricted to coastal waters less than 50 m deep, it plays an important role in regulating populations of fish and other marine animals of this ecosystem [32].**

*Comment. 3. Line 84: Few description of under Construction Bridge should be included like, how much area was covered by the pillars of the bridge, what was the distance between the two pillars during construction? etc.*

**R: We added the following sentences: “During the construction of the bridge, earthworks were carried out to facilitate the construction of the main pillar. The width of the estuary mouth was reduced, and the sandbank expanded (Fig 1). The reinforced concrete base (44 x 14 m) of the main pillar is the only submerged support remaining after the earthworks were removed at the end of the bridge construction.”**

*Comment. 4. Line 85: During the construction of the bridge, the width of the estuary mouth was reduced, and the sandbank expanded. This study was conducted in the estuary; have the any tidal affect observed in the behaviors of dolphin population during the study period? Or what were the visiting / sighting frequencies during the variation in the depth of the estuary.*

**R: We did not analyze the effect of tides in this study but added information on this topic in the discussion:** “A study conducted in 2006 in Pontal bay [31] concluded that dolphins were more frequent in areas deeper than 3 m and at the end of the flood, adapting the direction of their feeding movements to the tide. Another study in the Caravelas river estuary (located approximately 400 km south of the study area) suggests that depth is the main limiting factor for habitat use by Guiana dolphins [57]”.

*Comment. 5 Mention the table number in the entire manuscript*

**R: “Table 2” is now quoted in the text along with the numerical results.**

*Comment. 6. Justify lines and correct some minor grammatical mistakes in the manuscript*

**We have made a second in-depth revision to avoid formatting and grammatical errors.**

**Response to reviewer #3 comments:**

*Few comments:*

*It appears that Guiana dolphins forage in shallow water and estuaries and therefore, sand banks created due to bridge construction and other barriers facilitated herding of fishes by dolphin towards the barrier. It is an opportunistic foraging behavoiur. However, it imperative to look at the long-term impact of such barriers and adopted behaviour on the dolphin.*

*Authors would like to add such prospectives in their introduction as well as discussion. This will help in formulating long term planning for systematic monitoring of such projects which affects the critical aquatic lives.*

**R: Thank you for your comments and suggestions. We have added several sentences to address the long-term impact of environmental change in the introduction “These changes may result from modifications in prey distribution and behavior, as well as the removal or creation of foraging opportunities due to siltation.” and in the discussion: “****As the geomorphological and sedimentological dynamics of Pontal Bay are unpredictable, only monitoring the growth of the sandbank and the presence of dolphins will reveal whether the new opportunistic feeding area identified in this study will continue to attract dolphins or whether they will have to forage in other areas.”. Potential long-term effects are outlined at the end of the discussion: “We highlight that Pontal Bay has suffered continuous silting due to the deposition of continental and oceanic sediments.” (…) “The Jorge Amado Bridge poses yet another threat that could reduce the depth and width of Pontal Bay over time, two environmental characteristics that could decrease habitat suitability for dolphins. A continuous monitoring of Guiana dolphins in this area is highly recommended to understand the long-term impacts and to proactively conserve this population.”**
