# Supplementary material for: Response of Guiana dolphins to the construction of a bridge in Ilhéus, Northeastern Brazil
Source: PLoS One. 2024 Nov 15;19(11):e0312476. doi: 10.1371/journal.pone.0312476 (PMC11567520; doi:10.1371/journal.pone.0312476)
Supplement: Attachment — Submitted filename: Response to Reviewers.docx [file pone.0312476.s005.docx]

Response to reviewers

# PONE-D-24-14912

Dear Ram Kumar:

We are grateful for the interest of the journal PLOS ONE' in publishing our manuscript. This revision note is a response to your two comments on the revised version of our manuscript, for your consideration.

Title: Actually I am not in favour of a title in Interrogation; Interrogation is more often used for direct manipulation/experimental study

However for space use by dolphins there are several determinants including river water, morphometric flow patterns etc. Authors themselves explains in the text as “However, a single variable hardly explains habitat selection by dolphins [e.g. 4, 32,49].” Thus I would suggest a better title that could reflect this kind of study as follow:

“Effect of bridge constructions on dolphin use of space: A case study of Guiana dolphins from Ilhéus, Northeastern Brazil' Or "Responses of Guiana dolphins from Ilhéus Northeastern Brazil to bridge constructions"

**As suggested, we have adopted an affirmative form for the title:** **Response of Guiana dolphins to the construction of a bridge in Ilhéus, Northeastern Brazil.**

I think earlier statement has been misconstrued  i.e. Can the Ms be correlated with habitat shift of dolphins during Covid-19 lock down i.e. in contrary to bridge construction work (disturbance) undisturbed regime also affect home range of these river cetaceans. I did not intend to mention the effect of Covid-19; as other studies have reported reduced human disturbance due to covid -19 lock down Some such explanation about bankward shifting of Gangetic dolphins have been provided in following paper: COVID-19 lockdown affects zooplankton community structure in dolphin appearing site of the River Ganga at Patna_26_1 August 2023Aquatic Ecosystem Health and Management 26(1):20-31 The comment does not suggest mentioning Covid -19 effects. Covid-19 did not directly affect any nonhuman animal, however the lockdown offered opportunity to study behavioural changes in wild lives in the absence of human activities. So the comment suggests Habitat shifting, space use/ food base etc. in relation to anthropogenic disturbances. I am still of the view that this will further strengthen the quality of the paper. Covid-19 lock down was considered as reduced anthropogenic pressure /activity /disturbance from river bank side and no motor boat activity, undoubtedly these are determinants of shifting of dolphin habitat. The suggested paper has (i) presented data on food base of dolphin and habitat shifting as this manuscript directly explains the new opportunistic feeding area attracting dolphins. (ii) a probable spatial redistribution of feeding sites in lack of human activities during the lock down . It may be kept in mind that the Covid -19 lockdown is just a means that facilitated negligible or lower human disturbance. Justifiably anthropogenic disturbance are the direct determinant not the Covid-19.

**We understood that your previous comment referred to the decrease in anthropogenic pressure resulting from the lockdown and not to any direct effects of COVID. Although analysing the influence of COVID-19 lockdown is certainly a promising direction for future research, our results are not influenced by the COVID-19 lockdown, because 'The sampling was completed prior to the COVID-19 lockdown in Ilhéus'. We added this sentence to the Data Collection section in the R1 revised version of the manuscript and detail the chronology of the events in our previous response to reviewers.**

**Consequently, we cannot comment about “a probable spatial redistribution of feeding sites in lack of human activities during the lock down” or any other change in the dolphins’ behaviour during the lockdown because we did not conduct visual monitoring of the dolphins in the area during the lockdown.** **It was forbidden precisely because of the confinement requirement.**
